# Effects of treadmill training on gait of elders with Parkinson's disease: a literature review

**DOI:** 10.31744/einstein_journal/2020RW5233

**Published:** 2020-11-18

**Authors:** Natália Mariana Silva Luna, Guilherme Carlos Brech, Alexandra Canonica, Rita de Cássia Ernandes, Danilo Sales Bocalini, Julia Maria D’Andréa Greve, Angélica Castilho Alonso

**Affiliations:** 1 Universidade de São Paulo Faculdade de Medicina Hospital das Clínicas São PauloSP Brazil Instituto de Ortopedia e Traumatologia, Hospital das Clínicas, Faculdade de Medicina, Universidade de São Paulo, São Paulo, SP, Brazil.; 2 Universidade São Judas Tadeu São PauloSP Brazil Universidade São Judas Tadeu, São Paulo, SP, Brazil.; 3 Universidade Federal do Espírito Santo VitóriaES Brazil Universidade Federal do Espírito Santo, Vitória, ES, Brazil.

**Keywords:** Parkinson disease/rehabilitation, Gait, Aged

## Abstract

Parkinson's disease is the second most common neurodegenerative disorder in old age. Aging process for elders with Parkinson's disease can induce gait disturbances with more functional disabilities than for elders without the disease. Treadmill training as a therapy has resulted in notable effects on the gait of patients with Parkinson's disease and may be a resource for geriatric neurological rehabilitation. This review aimed to study the effects on gait after treadmill training in elderly patients with Parkinson's disease. The search was performed in the databases PubMed®, LILACS, PEDro and EMBASE, with the following keywords: “Parkinson's disease”, “elderly”, “treadmill training” and “gait evaluation”. The quality of the studies included was assessed by PEDro Scale. Eleven studies met the inclusion and exclusion criteria. Eight studies were randomized, and only one did a follow-up. One can observe in this review that treadmill training with or without weight support (at least 20 minutes, two to three times a week, with progressive increase of loads, for minimum of 6 weeks) in elderly patients with the Parkinson's disease was effective to improve gait. In addition, both were considered safe (since some studies described the use of belts, even in unsupported training) and can be associated with therapies complementary to gait, such as repetitive transcranial magnetic stimulation, visual cues or anodal transcranial direct current stimulation. Treadmill training in elderly patients with Parkinson's disease is an intervention that improves gait outcomes, but further studies are required for better proofs.

## INTRODUCTION

Idiopathic Parkinson's disease (PD) is a chronic, progressive and degenerative disease of the central nervous system, associated with a depletion in dopaminergic neurons in the nigrostriatal pathway.^(^^1^^)^ Clinical symptoms appear when there is a 40% to 60% reduction of nigral neurones and striatal dopamine.^(^^2^^)^ Parkinson's disease is the second most common degenerative nervous system disease, affecting 1% to 2% of the population older than 65 years.^(^^3^^)^ The proportion of elderly in the world is increasing, resulting in larger pool of people at risk for PD.^(^^4^^)^

Cardinal symptoms include resting tremor, muscular rigidity, freezing of gait (FOG); bradykinesia and gait and postural instability.^(^^3^^,^^4^^)^ Gait disorders compromise patients’ independence and quality of life, decreasing mobility and increasing the risk of falls and fractures.^(^^5^^)^Parkinson's disease induces gait disturbances, such as reduced velocity, reduced stride and step length, and increased duration of double stance.^(^^4^^–^^8^^)^

The reduction in gait speed is explained by slower center of gravity displacement, decreased vertical component of ground reaction force^(^^9^^–^^11^^)^ and greater lateral displacement of the center of pressure at the beginning of gait.^(^^12^^)^ The reduction in step length occurs mainly due to the deficiency in the detachment phase of the foot, in which a smaller electromyography signal in the gastrocnemius muscle is observed and consequently problems to project limb forwards.^(^^10^^)^ Increased support phase is related to decreased gait speed and bradykinesia.^(^^5^^)^ This hinders the automatism of the step during the gait and promotes electromyographic signal changes of the lower limbs.^(^^13^^,^^14^^)^

Similarly, aging also slows gait velocity and step length, which suggests that PD in elders may lead to greater functional disability, making them more susceptible to falls.^(^^15^^)^

Concerning physical gait rehabilitation, treadmill training (TT) as a therapy has shown notable effects on the gait of patients with PD, with superior results in gait parameters compared to patients not undergoing this treatment.^(^^16^^–^^21^^)^ Literature reviews show that the TT can promote a more stable walking pattern in PD patients, and suggest that the intervention is able to restore rhythmicity and reduces gait variability.^(^^18^^,^^21^^,^^22^^)^ However, they analyzed various outcomes, but with no emphasis on the elderly. Hence, the objective of this work was to review the effects on gait after TT in elderly PD patients.

## OBJETIVE

The objective of this study was to review the effects on gait after treadmill training in elderly Parkinson's disease patients.

## METHODS

### Search strategy

The search was performed in PubMed®, LILACS, PEDro and EMBASE database; and published articles related to the effects of TT on the gait of elderly patients with PD were found based on the following keywords: “Parkinson's disease”, “elderly”, “treadmill training” and “gait evaluation”.

### Study selection

The critical literature review identified the following inclusion criteria: studies in humans, in English, and published in scientific journals during the last 10 years. Articles describing patients aged less than 60 years, or who have not undergone treadmill gait training, and without outcome measures regarding gait were excluded. Articles describing treadmill gait training in patients without PD and treadmill gait training in combination with virtual reality were also excluded. After reading the titles and/or abstracts, articles meeting the inclusion criteria and identified as relevant to the development of this work were selected. A total of 25 references to simple combinations were found in PubMed®, LILACS, PEDro and EMBASE. Eleven articles were finally selected based on the established criteria.

### Quality appraisal

The quality of 11 studies included^(^^23^^–^^33^^)^ was assessed by PEDro scale checklist ( Table 1 ), which presents 11 items that consider the following criteria: eligibility criteria and source of participants; random allocation; blind allocation; comparability of baseline; subjects were blind; therapists were blind; evaluators were blind; results with more than 85% of participants; intention-to-treat analysis; statistical comparisons between groups; and point measures and variability measures.

**Table 1:** PEDro scale of the 11 studies selected

Authors	Eligibility	Random allocation	Blind allocation	Comparability of baseline	Subjects were blind	Therapists were blind	Evaluators were blind	Results with more than 85% of participants	Intention-to-treat analysis	Statistical comparisons between groups	Point measures and variability measures
Frazzitta et al.^(^^23^^)^	Yes	Yes	No	Yes	No	No	Yes	Yes	Yes	Yes	No
Yang et al.^(^^24^^)^	Yes	No	Yes	Yes	Yes	Yes	Yes	Yes	Yes	Yes	Yes
Galli et al.^(^^25^^)^	Yes	No	Yes	Yes	Yes	Yes	Yes	Yes	Yes	Yes	No
Canning et al.^(^^26^^)^	Yes	No	Yes	Yes	No	No	Yes	Yes	Yes	Yes	Yes
Frazzitta et al.^(^^27^^)^	Yes	Yes	no	Yes	No	No	Yes	Yes	Yes	Yes	No
Picelli et al.^(^^28^^)^	Yes	No	Yes	Yes	No	No	Yes	Yes	Yes	Yes	No
Fisher et al.^(^^29^^)^	Yes	No	Yes	Yes	Yes	No	No	Yes	Yes	Yes	No
Schlick et al.^(^^30^^)^	Yes	No	Yes	Yes	No	No	No	Yes	Yes	Yes	No
Arcolin et al.^(^^31^^)^	Yes	No	No	Yes	No	No	No	Yes	Yes	Yes	No
Herman et al.^(^^32^^)^	Yes	No	No	No	No	No	No	Yes	Yes	No	No
Fernández-Lago et al.^(^^33^^)^	Yes	No	No	Yes	No	No	No	Yes	Yes	Yes	No

## RESULTS

Eleven studies met the inclusion criteria.^(^^23^^–^^33^^)^ Studies that obtained the highest score on the PEDro scale were conducted by Yang et al.,^(^^24^^)^ and Galli el al.,^(^^25^^)^ ( Table 1 ).

Of the 11 studies,^(^^23^^–^^33^^)^ eight were randomized.^(^^23^^–^^30^^)^ In relation the type of training, five studies analyzed training without body weight support,^(^^23^^,^^24^^,^^26^^,^^27^^,^^30^^)^ and three studies evaluated with support system^(^^25^^,^^28^^,^^29^^)^ ( Table 2 ).

**Table 2:** Summary of the population, protocols, gait measures and results of these investigations

Studies	Population	Protocol	Gait measures	Results
Frazzitta et al.^(^^23^^)^	30 patients with gait disturbance, but without FOG in “on” stage and 30 patients with FOG in “on” stage	Patients underwent a 4-week rehabilitation treatment using a treadmill with auditory and visual cues	Gait speed, stride length, asymmetry of gait, 6MWT, UPDRS II-III, Berg Balance Scale, TUG, comfortable-fast gait speeds, FOG-Q	Data support a direct involvement of the asymmetry of gait in the development of FOG in PD TT is effective in improving gait and balance in PD FOG patients, and this might be related to a reduction of asymmetric gait
Yang et al.^(^^24^^)^	20 patients with PD with ability to walk independently	-2 groups: 1. Participants received rTMS (Experimental Group) 2. Participants received sham rTMS (Control Group) Followed by TT (30 minutes) for 12 sessions over 4 weeks (3 sessions a week)	Corticomotor inhibition and walking performance	The findings suggested that combination of rTMS and TT enhances the effect of TT on modulation of corticomotor inhibition and improvement of walking performance in those with PD
Galli et al.^(^^25^^)^	50 idiopathic PD Ability to walk, unassisted or with little assistance, for a distance of 25 feet	-2 groups 1. Robot-assisted therapy group (n=25) 2. Intensive treadmill therapy group (n=25)	Hoehn and Yahr scaleUPDRS Optoelectronic system (ELITE2002, BTS, Milan, Italy)	In the robot group, differences were found in kinematic variables (pelvic obliquity and hip abduction and adduction). The intensive group showed no statistically significant changes. The end-effector robotic rehabilitation locomotor training improved gait kinematics and seems to be effective for rehabilitation in patients with mild PD
Canning et al.^(^^26^^)^	20 people with idiopathic PD with <2 hours per week of leisure time physical activity in prior 3 months, and had a stable response to levodopa medications	-2 groups: 1. The Experimental Group undertook a semi-supervised home-based program of 30-40 minutes of treadmill walking, 4 times a week for 6 weeks 2. The Control Group received usual care (i.e. advice to maintain current levels of physical activity)	The primary outcome measure of efficacy was walking capacity (6MWT)	Semi-supervised home-based TT is a feasible and safe form of exercise for cognitively intact people, with mild PD
Frazzita et al.^(^^27^^)^	40 Parkinsonian patients with freezing were randomly assigned to two groups	2 group: 1. Underwent a rehabilitation program based on treadmill training associated with auditory and visual cues 2. Followed a rehabilitation protocol using cues and not associated with treadmill	Functional evaluation was based on the Unified Parkinson's Disease Rating Scale Motor Section (UPDRS III); Freezing of Gait Questionnaire (FOGQ), 6-minute walking test (6MWT), gait speed, and stride cycle	The results suggest that treadmill training associated with auditory and visual cues might give better results than more conventional treatments. Treadmill training probably acts as a supplementary external cue
Picelli et al.^(^^28^^)^	60 patients with mild to moderate PD	3 groups: 1. Robotic gait training group (n=20);TT group (n=20) performed equal intensity 2. TT without body-weight support 3. The physical therapy group (n=20) underwent conventional gait therapy45 minutes per session 3 days/week, during four consecutive weeks	10-minute walking test6MWT	No statistically significant difference was found between the robotic gait training group and the TT group in the evaluation after training. Statistically significant improvement was found after treatment in favor of the robotic gait training group and TT group compared to the physical therapy group. Findings were confirmed at the 3-month follow-up evaluation
Fisher et al.^(^^29^^)^	30 PD patients, within 3 years of diagnosis, with Hoehn and Yahr stages 1 or 2	3 groups: 1. High-intensity (body weight-supported TT) 2. Low-intensity exercise (exercises to range of motion, balance and gait) 3. Zero-intensity (education group)	UPDRS Biomechanical analysis of self-selected and fast walking Sit-to-stand tasks Corticomotor excitability	A small improvement in total and motor UPDRS was observed in all groups. High-intensity group subjects showed post-exercise increases in gait speed, step and stride length, and hip and ankle joint excursion during self-selected and fast gait, besides improved weight distribution during sit-to-stand tasks. Improvements in gait and sit-to-stand measures were not consistently observed in the low- and zero-intensity groups. The high-intensity group showed lengthening in cortical silent period
Schlick et al.^(^^30^^)^	23 outpatients with PD	Patients received 12 training sessions within 5 weeks of either visual cues combined with TT (n=12) or pure TT (n=11)	Gait speed, stride length and cadence Functional tests included the TUG test, the UPDPRS and the freezing of gait-questionnaire	This pilot study suggests that visual cues combined with TT have more beneficial effects on gait than pure TT, in patients with a moderate stage of PD. A large-scale study with longer follow-up is required
Arcolin et al.^(^^31^^)^	29 patients with the diagnosis of idiopathic PDAbility to walk, unassisted	2 groups (randomized): 1. Treadmill (n=13) 2. Cycle ergometer (n=16) Training for 3 weeks, 1 hour/day	6MWT Spatiotemporal variables of gait assessed by baropodometry TUG test MINIBESTest UPDRS	This pilot study shows that cycle ergometer training improves walking parameters and reduces clinical signs of PD, as much as TT does. Gait velocity is accompanied by step lengthening, making the gait pattern close to that of healthy subjects. Cycle ergometer is a valid alternative to treadmill for improving gait in short-term in patients with PD
Herman et al.^(^^32^^)^	9 patients with PD who were able to ambulate independently and were not demented	Patients walked on a treadmill for 30 minutes during each training session, 4 training sessions a week, for 6 weeks	PDQ-39 UPDRS Gait speed, stride time variability, swing time variability SPPB	These results show the potential to enhance gait rhythmicity in patients with PD and suggest that a progressive and intensive TT program can be used to minimize impairments in gait, reduce fall risk, and increase quality of life of these patients
Fernández-Lago et al.^(^^33^^)^	18 idiopathic PD patientsAbility to walk, unassisted	18 participants with PD were evaluated under the following three conditions (groups): 1. Treadmill walking alone (treadmill) 2. Treadmill walking combined with anodal tDCS (AtDCS + treadmill) delivered over the motor cortex 3. Treadmill walking combined with sham stimulation (StDCS+treadmill)	Overground walking performance, soleus H-reflex, reciprocal Ia inhibition from the tibialis anterior to the soleus muscle, intracortical facilitation, and short intracortical inhibition of the tibialis anterior muscle	All treadmill conditions improved walking performance and modulated spinal and corticospinal parameters compare with other kind of treatments. However, AtDCS + treadmill lead to a different modulation of reciprocal Ia inhibition in comparison with the other treadmill conditions

FOG: freezing of gait; 6MWT: 6-Minute Walk Test; UPDPRS: Unified Parkinson Disease Rating Scale; TUG: Timed Up and Go; FOG-Q: Freezing of Gait Questionnaire; TT: treadmill training; PD: Parkinson disease; rTMS: repetitive transcranial magnetic stimulation; MINIBESTest: Balance Evaluation Systems Test; PDQ-39: Parkinson Disease Questionnaire-39; SPPB: Short Physical Performance Battery; tDCS: transcranial direct current stimulation.

## DISCUSSION

Some studies on TT in PD patients and literature reviews address the subject. However, the reviews analyzed all age groups and present methodologies with different outcomes: quality of life, balance and gait. Our review aimed to focus only studies with groups of PD patients older than 60 years, as well as outcomes related gait specificities; and in according with check list of PEDro scale, the articles included were considered adequate for scientific publication.

The Hoehn and Yarh scale ranged from 1 to 3, with the most^(^^23^^,^^25^^–^^27^^,^^29^^,^^32^^,^^33^^)^ addressed patients between 1 and 2, a score that represents discrete balance impairment. In four studies^(^^24^^,^^28^^,^^30^^,^^31^^)^ the stage was between 2 to 3, a score in which the disease is necessarily bilateral and exists some postural instability, but the patient is physically independent.

The Hoehn and Yarh scale characterizes the patient's functional status through observation.^(^^34^^)^ Other studies with patients younger than 60 years or mixing different age groups also showed this Hoehn and Yarh scale score. Thus, the moderate Hoehn and Yarh score could suggest aging did not interfere in the disease.

The duration of disease was another demographic variable that was different among the studies. In eight studies,^(^^15^^–^^17^^,^^19^^,^^21^^–^^24^^)^ the duration was between 5 and 10 years and, in two, the duration was more than 10 years.^(^^27^^,^^29^^)^ Only one did not mention this data.^(^^23^^)^ Studies showed that, with the evolution of the disease (5 to 7 years), there is worsening of motor condition and onset of dyskinesias.^(^^15^^)^ Seventy percent of patients taking levodopa for 6 or more years have motor complications and need to discontinue use temporarily (“off”).^(^^35^^)^

The gait evaluations used in studies of review were computerized or functional. Both are important for the explanations of results, but some are more specific than others and thus may help more for the answers on clinical practice.^(^^11^^)^

The motor score of Unified Scale for Parkinson's Disease (UPDRS) was used in most articles.^(^^23^^,^^29^^–^^32^^)^It is a very reliable (r=0.96) and valid scale, a suitable method for evaluating PD.^(^^36^^)^ The motor exploration section consists of 14 items (numbers ranging from 18 to 31), but the item that evaluates gait is observational and does not allow accurate analysis.

The Timed Up and Go test (TUG) was also used in five studies.^(^^23^^,^^27^^,^^30^^–^^32^^)^ The TUG measures the time ofmotor task of to get up from chair, to walk 3m, to return and to sit.^(^^37^^)^ Isles et al.,^(^^38^^)^ define the test as sensitive and specific to discriminate between fallers and non-faller. It is possible to observe that the test is considered for balance and mobility,^(^^39^^)^ without, however, analyzing gait specific characteristics.

Another evaluation tool used was the 6-minute walk test (6MWT).^(^^14^^,^^16^^–^^19^^)^ It is used to evaluate physical response and provides comprehensive analysis of respiratory, cardiac and metabolic systems.^(^^40^^)^ The test reflects limitations of daily life activities, such as gait, but according to the American Thoracic Society (ATS),^(^^41^^)^ the accurate indication of 6MWT is the presence of pulmonary disease or mild to moderate heart disease, so that the test is used to measure response to treatment and to predict morbidity and mortality.

Spatial parameters (velocity, step length and cadence) are considered significant for gait clinical evaluation. They can be calculated qualitatively, without cameras and software, or computerized by kinematic and kinetic analyses. Five studies investigated these parameters through qualitative approach^(^^27^^,^^29^^–^^32^^)^ and only two by computerized means.^(^^25^^,^^29^^)^ The use of three-dimensional gait analysis is scarce in studies about PD, but is considered as gold standard method to better biomechanical understanding, providing reproducible and reliable data.^(^^13^^)^

The TT was studied with and without body weight support. Eight studies analyzed training without body weight support,^(^^14^^–^^18^^,^^22^^–^^24^^)^ and three studies assessed with support system.^(^^19^^–^^21^^)^ All showed improvements in gait variables after training, but there were differences between the methodologies.

Only Herman et al.,^(^^32^^)^ did a follow-up study. They performed 6 weeks of TT (four times a week, 30 minutes each session), followed by 1 month of TT with progressive increase of intensity in nine patients with PD. There was improvement of UPDRS regarding velocity and step length, as well as gait variability after training period and follow-up of 6 weeks. These results suggested that a progressive and intensive TT program can be used to minimize impairments in gait, reduce fall risk, and increase quality of life in these patients.

Frazzitta et al.,^(^^23^^)^ recruited Parkinson's patients with and without FOG, who underwent the same protocol, TT without support associated with visual and auditory cues. Subjects received TT for 30 minutes every day for 5 days a week, during 4 weeks (20 sessions in total). Treadmill training is effective in improving gait and balance in PD patients and this asymmetry in gait may be related to an increased chance of FOG.

The others compared the traditional TT with other type of TT. Frazzitta et al.,^(^^27^^)^ compared TT without support associated with visual and auditory cues with gait training without treadmill, also associated with visual and auditory cues. The results suggest that TT associated with auditory and visual cues might give better results than more conventional treatments. Subjects received training for 20 minutes every day, for 4 weeks (28 sessions in all). Treadmill training probably acts as a supplementary external cue.

Schlick et al.,^(^^30^^)^ analyzed 20 patients with PD, who were divided into two interventions: TT group with visual cues and TT group no cues. This pilot study suggests that visual cues combined with TT have more beneficial effects on gait than traditional TT. The visual cue acts as a stimulus to visual receptors, generating repetitive sensory afferent stimuli to the central nervous system. In PD patients these signals may also represent external sensory cues, a trigger for intact circuits unaffected by the disease, such as the lateral pathway of the premotor cortex.^(^^2^^,^^6^^)^

Yang et al.,^(^^24^^)^ compared TT with and without prior application of repetitive transcranial magnetic stimulation (rTMS). The TT was carried out for 12 sessions (3 sessions a week) over a 4-week period. All participants walked on a motorized treadmill (Biodex, Shirley, New York, USA) after the rTMS. The findings suggested that combination of rTMS and TT enhances the effect of TT on modulation of corticomotor inhibition and improvement of walking performance in PD patients.

Canning et al.,^(^^26^^)^ observed an Experimental Group undertook a semi-supervised home-based program of 30 to 40 minutes of treadmill walking, four times a week for 6 weeks. The Control Group received usual care ( *i.e* . advice to maintain current levels of physical activity). Semi-supervised home-based TT is a feasible and safe form of exercise for cognitively intact people with mild PD. The home treatment is an important strategy in geriatric rehabilitation, and it is possible to associate TT as a reinforcement to gait exercises.

Arcolin et al.,^(^^31^^)^ randomized 29 PD inpatients to treadmill (n=13) or cycle ergometer (n=16) training for 3 weeks, 1 hour per day. This pilot study shows that cycle ergometer training improves walking parameters and reduces clinical signs of PD, as much as TT does. Gait velocity is accompanied by step lengthening, making the gait pattern close to that of healthy subjects. Cycle ergometer is a valid alternative to treadmill for improving gait in short term in patients with PD.

Studies not included in review due to sample with no elderly also showed that TT without body weight support in patients with PD, compared to other motor therapies, increased speed and stride length.^(^^42^^,^^43^^)^

Three studies included used TT with body weight support^(^^18^^–^^20^^)^ and two^(^^19^^,^^21^^)^ showed superiority of supported training compared to other therapy, and one did not observe a difference.^(^^28^^)^ Fisher et al.,^(^^29^^)^ recruited 30 PD patients (stage Hoehn and Yarh I to II) who were randomized into three groups: high-intensity therapy (supported training), low-intensity physical therapy (passive and active range of motion, balance, gait, muscular endurance and daily functions exercises) and non-intensive therapy, characterized by therapeutic education (six classes during a 8-week period about quality of life). The interventions lasted 8 weeks, totaling 24 sessions (three times a week with 1-hour sessions). The TT started with 10% of suspended body weight, however if the patient could not perform the gait, this percentage was increased, so that the goal of each session was for the patient to reach and maintain a level higher than 3 METS, while respecting rest, whenever necessary. The results showed improvement in UPDRS in all groups. The supported training group showed increase in gait velocity, step and stride length, and excursions of hip and ankle in sagittal plane, as well as improved weight distribution during exercise and raise of cortical silent period.

Gali et al.,^(^^25^^)^ and Picelli et al.,^(^^28^^)^ also used training with body weight support, however with robotic assistance. Picelli et al.,^(^^28^^)^ compared three groups: the robotic gait training group; TT without body-weight support group and physical therapy group. Each training session consisted of three parts, with a 5-minute rest after each. First, we trained patients at 1km/hour of speed for 10 minutes; then, at 1.5km/hour of speed for 10 minutes; finally, at 2.0km/hour of speed for 10 minutes. There were no differences in the spatial and temporal parameters of the 6- and 10-minute walk tests. Gali et al.,^(^^25^^)^ evaluated 50 PD patients, who were divided into two groups: 25 were assigned to the robot-assisted therapy and 25 to the intensive treadmill therapy group. The end-effector robotic rehabilitation locomotor training improved gait kinematics, and seems to be effective for rehabilitation in patients with mild PD.

Some studies with non-elderly patients also observed similar results. There were no differences in temporal-spatial variables of the 6-minute and 10-minute walk tests and TUGT and UPDRS,^(^^44^^)^ when comparison between TT and Lokomat TT was performed. Other studies have observed the effect of TT with weight support in relation to without weight support,^(^^45^^)^ to ground gait training^(^^46^^)^ and to other motor therapies.^(^^16^^,^^17^^)^

Toole et al.,^(^^45^^)^ reported the effects of TT for 6 weeks, on 23 PD patients randomized into three groups. Two groups trained gait with 25% and 5% of body weight support, and the third group without support. The three groups improved on the UPDRS and Berg scales, with no difference between them. The study by Ganesan et al.,^(^^46^^)^ used computerized posturography to evaluate the effect of a 4-week TT with 20% body weight support, four times a week (30 minutes per day). The authors reported improvements in the limits of stability and medial lateral oscillation of the center of mass with the training with support in relation to the training of conventional gait off the treadmill. Miyai et al.,^(^^16^^)^ compared gait training with 20% body weight support *versus* conventional physiotherapy for 4 weeks in PD patients, and reported that weight-bearing TT improved gait motor performance (10-minute walk test) and in daily life activities, which persisted for 6 months.^(^^17^^)^

Fernández-Lago et al.,^(^^33^^)^ described that a single session combining treadmill walking and anodal transcranial direct current stimulation (tDCS) delivered over the motor cortex resulted in a specific modulation of the reciprocal a inhibition from the tibialis anterior to the soleus muscle. However, this acute effect did not result in improvements of gait parameters associated with treadmill walking in Parkinson's disease.

Unsupported body weight training seems to be more challenging because it requires greater muscle demand. The main reasons for this evidence are constant velocity; continuous sensory stimulation; external sensory cues; activation of gait neural circuits generating central pattern; visual feedback; constant speed and motor learning.^(^^16^^,^^17^^,^^45^^,^^46^^)^

Training with body weight support, even if it decreases peripheral stimulation (muscle strength and aerobic effort), may increase proprioceptive stimulation.^(^^47^^)^The partial weight support can also extend the useful training period because it reduces fatigue.^(^^48^^)^ In addition, facilitates movement of lower limbs,^(^^49^^)^ and can therefore be considered a promising intervention for sensorimotor control training by improvement of postural reflexes.^(^^16^^,^^17^^)^ It may also facilitate the use of strategies that use non-dopaminergic pathways and are not affected by disease. Other authors suggested that training with weight support stimulated the gait control in spinal cord.^(^^17^^,^^47^^)^

It is possible to observe in review that TT with or without weight support (at least 20 minutes, 2 to 3 times a week, with progressive increase of loads, for a minimum of 6 weeks) in elderly PD patients is effective for improving gait. In addition, both are considered safe (since even unsupported training, studies describe the use of belts) and can be associated with therapies complementary to gait, such as rTMS, visual cues or anodal tDCS.

However, it is important to emphasize that assessments used to gait were different. Five studies^(^^17^^,^^19^^,^^21^^,^^22^^,^^24^^)^ were able to evaluate gait clinical characteristics, which can help in evolution of treatment. Only two used gold standard measure, the computerized evaluation.^(^^25^^,^^29^^)^ Treadmill training with or without weight support can be an intervention to neurological geriatric rehabilitation, but there is a need for more studies with this theme that better characterize the sample of elderly with PD, as well as using more specific gait assessment tools.

There are some limitations in this study. We did not analyze the subgroups of elderly patients among the studies that recruited adults in the sample and, therefore, were excluded. Most of the studies have a relatively small sample, with a total of 300 patients in all studies.

## CONCLUSION

Treadmill training with or without weight support (at least 20 minutes, two to three times a week, with progressive increase of loads, for minimum of 6 weeks) is an intervention that improves gait outcomes in elderly Parkinson's disease patients, but further studies are warranted.

## References

[1] 1. Pieruccini-Faria F, Menuchi MR, Vitório R, Gobbi LT, Stella F, Gobbi S. Parâmetros cinemáticos da marcha com obstáculos em idosos com Doença de Parkinson, com e sem efeito da levodopa: um estudo piloto. Rev Bras Fisioter. 2006;10(2):233-9.

[2] 2. de Goede CJ, Keus SH, Kwakkel G, Wagenaar RC. The effects of physical therapy in Parkinson's disease: a research synthesis. Arch Phys Med Rehabil. 2001;82(4):509-15. 10.1053/apmr.2001.22352 11295012

[3] 3. Svehlík M, Zwick EB, Steinwender G, Linhart WE, Schwingenschuh P, Katschnig P, et al. Gait analysis in patients with Parkinson's disease off dopaminergic therapy. Arch Phys Med Rehabil. 2009;90(11):1880-6. 10.1016/j.apmr.2009.06.017 19887212

[4] 4. Blin O, Ferrandez AM, Serratrice G. Quantitative analysis of gait in Parkinson patients: increased variability of stride length. J Neurol Sci. 1990;98(1):91-7. 10.1016/0022-510x(90)90184-o 2230833

[5] 5. Peppe A, Chiavalon C, Pasqualetti P, Crovato D, Caltagirone C. Does gait analysis quantify motor rehabilitation efficacy in Parkinson's disease patients? Gait Posture. 2007;26(3):452-62. 10.1016/j.gaitpost.2006.11.207 17240143

[6] 6. Morris ME, Iansek R, Matyas TA, Summers JJ. The pathogenesis of gait hypokinesia in Parkinson's disease. Brain. 1994;117(Pt 5):1169-81. 10.1093/brain/117.5.1169 7953597

[7] 7. Morris ME, Iansek R, Matyas TA, Summers JJ. Stride length regulation in Parkinson's disease. Normalization strategies and underlying mechanisms. Brain. 1996;119(Pt 2):551-68. 10.1093/brain/119.2.551 8800948

[8] 8. Camicioli R, Oken BS, Sexton G, Kaye JA, Nutt JG. Verbal fluency task affects gait in Parkinson's disease with motor freezing. J Geriatr Psychiatry Neurol. 1998;11(4):181-5. 10.1177/089198879901100403 10230996

[9] 9. Galletly R, Brauer SG. Does the type of concurrent task affect preferred and cued gait in people with Parkinson's disease? Aust J Physiother. 2005;51(3):175-80. 10.1016/s0004-9514(05)70024-6 16137243

[10] 10. Sofuwa O, Nieuwboer A, Desloovere K, Willems AM, Chavret F, Jonkers I. Quantitative gait analysis in Parkinson's disease: comparison with a healthy control group. Arch Phys Med Rehabil. 2005;86(5):1007-13. 10.1016/j.apmr.2004.08.012 15895349

[11] 11. Liu W, McIntire K, Kim SH, Zhang J, Dascalos S, Lyons KE, et al. Bilateral subthalamic stimulation improves gait initiation in patients with Parkinson's disease. Gait Posture. 2006;23(4):492-8. 10.1016/j.gaitpost.2005.06.012 16098748

[12] 12. Gantchev N, Viallet F, Aurenty R, Massion J. Impairment of posturo-kinetic co-ordination during initiation of forward oriented stepping movements in parkinsonian patients. Electroencephalogr Clin Neurophysiol. 1996;101(2):110-20. 10.1016/0924-980x(95)00253-h 8647016

[13] 13. Roiz RM, Cacho EW, Pazinatto MM, Reis JG, Cliquet A Jr, Barasnevicius-Quagliato EM. Gait analysis comparing Parkinson's disease with healthy elderly subjects. Arq Neuropsiquiatr. 2010;68(1):81-6. 10.1590/s0004-282x2010000100018 20339659

[14] 14. McVey MA, Stylianou AP, Luchies CW, Lyons KE, Pahwa R, Jernigan S, et al. Early biomechanical markers of postural instability in Parkinson's disease. Gait Posture. 2009;30(4):538-42. 10.1016/j.gaitpost.2009.08.232 19748271

[15] 15. Braak H, Del Tredici K, Rüb U, de Vos RA, Jansen Steur EN, Braak E. Staging of brain pathology related to sporadic Parkinson's disease. Neurobiol Aging. 2003;24(2):197-211. 10.1016/s0197-4580(02)00065-9 12498954

[16] 16. Miyai I, Fujimoto Y, Ueda Y, Yamamoto H, Nozaki S, Saito T, et al. Treadmill training with body weight support: its effect on Parkinson's disease. Arch Phys Med Rehabil. 2000;81(7):849-52. 10.1053/apmr.2000.4439 10895994

[17] 17. Miyai I, Fujimoto Y, Yamamoto H, Ueda Y, Saito T, Nozaki S, et al. Long-term effect of body weight-supported treadmill training in Parkinson's disease: a randomized controlled trial. Arch Phys Med Rehabil. 2002;83(10):1370-3. 10.1053/apmr.2002.34603 12370870

[18] 18. Herman T, Giladi N, Hausdorff JM. Treadmill training for the treatment of gait disturbances in people with Parkinson's disease: a mini-review. J Neural Transm (Vienna). 2009;116(3):307-18. Review. 10.1007/s00702-008-0139-z 18982238

[19] 19. Hottenrott K, Ludyga S, Schulze S. Effects of high intensity training and continuous endurance training on aerobic capacity and body composition in recreationally active runners. J Sport Sci Med. 2012;11(3):483-8. PMC3737930 24149357

[20] 20. Schenkman M, Hall DA, Barón AE, Schwartz RS, Mettler P, Kohrt WM. Exercise for people in early- or mid-stage Parkinson disease: a 16-month randomized controlled trial. Phys Ther. 2012;92(11):1395-410. 10.2522/ptj.20110472 PMC3488266 22822237

[21] 21. Bello O, Fernandez-Del-Olmo M. How does the treadmill affect gait in Parkinson's disease? Curr Aging Sci. 2012;5(1):28-34. Review. 10.2174/1874609811205010028 21762092

[22] 22. Mehrholz J, Kugler J, Storch A, Pohl M, Hirsch K, Elsner B. Treadmill training for patients with Parkinson's disease. Cochrane Database Syst Rev. 2015;(8):CD007830. Update in: Cochrane Database Syst Rev. 2015; (9):CD007830. 10.1002/14651858.CD007830.pub4 PMC9579750 26363646

[23] 23. Frazzitta G, Pezzoli G, Bertotti G, Maestri R. Asymmetry and freezing of gait in parkinsonian patients. J Neurol. 2013;260(1):71-6. 10.1007/s00415-012-6585-4 22752088

[24] 24. Yang YR, Tseng CY, Chiou SY, Liao KK, Cheng SJ, Lai KL, et al. Combination of rTMS and treadmill training modulates corticomotor inhibition and improves walking in Parkinson disease: a randomized trial. Neurorehabil Neural Repair. 2013;27(1):79-86. 10.1177/1545968312451915 22785003

[25] 25. Galli M, Cimolin V, De Pandis MF, Le Pera D, Sova I, Albertini G, et al. Robot-assisted gait training versus treadmill training in patients with Parkinson's disease: a kinematic evaluation with gait profile score. Funct Neurol. 2016;31(3):163-70. 10.11138/FNeur/2016.31.3.163 PMC5115231 27678210

[26] 26. Canning CG, Allen NE, Dean CM, Goh L, Fung VS. Home-based treadmill training for individuals with Parkinson's disease: a randomized controlled pilot trial. Clin Rehabil. 2012;26(9):817-26. 10.1177/0269215511432652 22257506

[27] 27. Frazzitta G, Maestri R, Uccellini D, Bertotti G, Abelli P. Rehabilitation treatment of gait in patients with Parkinson's disease with freezing: a comparison between two physical therapy protocols using visual and auditory cues with or without treadmill training. Mov Disord. 2009;24(8):1139-43. 10.1002/mds.22491 19370729

[28] 28. Picelli A, Melotti C, Origano F, Neri R, Waldner A, Smania N. Robot-assisted gait training versus equal intensity treadmill training in patients with mild to moderate Parkinson's disease: a randomized controlled trial. Parkinsonism Relat Disord. 2013;19(6):605-10. 10.1016/j.parkreldis.2013.02.010 23490463

[29] 29. Fisher BE, Wu AD, Salem GJ, Song J, Lin CH, Yip J, et al. The effect of exercise training in improving motor performance and corticomotor excitability in people with early Parkinson's disease. Arch Phys Med Rehabil. 2008;89(7):1221-9. 10.1016/j.apmr.2008.01.013 PMC2989816 18534554

[30] 30. Schlick C, Ernst A, Bötzel K, Plate A, Pelykh O, Ilmberger J. Visual cues combined with treadmill training to improve gait performance in Parkinson's disease: a pilot randomized controlled trial. Clin Rehabil. 2016;30(5):463-71. 10.1177/0269215515588836 26038610

[31] 31. Arcolin I, Pisano F, Delconte C, Godi M, Schieppati M, Mezzani A, et al. Intensive cycle ergometer training improves gait speed and endurance in patients with Parkinson's disease: a comparison with treadmill training. Restor Neurol Neurosci. 2016;34(1):125-38. 10.3233/RNN-150506 26684265

[32] 32. Herman T, Giladi N, Gruendlinger L, Hausdorff JM. Six weeks of intensive treadmill training improves gait and quality of life in patients with Parkinson's disease: a pilot study. Arch Phys Med Rehabil. 2007;88(9):1154-8. 10.1016/j.apmr.2007.05.015 17826461

[33] 33. Fernández-Lago H, Bello O, Mora-Cerdá F, Montero-Cámara J, Fernández-Del-Olmo MÁ. Treadmill walking combined with anodal transcranial direct current stimulation in Parkinson disease: a pilot study of kinematic and neurophysiological effects. Am J Phys Med Rehabil. 2017;96(11):801-8. 10.1097/PHM.0000000000000751 28398968

[34] 34. Hoehn MM, Yahr MD. Parkinsonism: onset, progression, and mortality. Neurology. 1967;17(5):427-42. 10.1212/wnl.17.5.427 6067254

[35] 35. Schapira AH, Emre M, Jenner P, Poewe W. Levodopa in the treatment of Parkinson's disease. Eur J Neurol. 2009;16(9):982-9. Review. 10.1111/j.1468-1331.2009.02697.x 19538218

[36] 36. Jenkins ME, Johnson AM, Holmes JD, Stephenson FF, Spaulding SJ. Predictive validity of the UPDRS postural stability score and the functional reach test, when compared with ecologically valid reaching tasks. Parkinsonism Relat Disord. 2010;16(6):409-11. 10.1016/j.parkreldis.2010.04.002 20434938

[37] 37. Barry E, Galvin R, Keogh C, Horgan F, Fahey T. Is the Timed Up and Go test a useful predictor of risk of falls in community dwelling older adults: a systematic review and meta-analysis. BMC Geriatr. 2014;14(1):14. Review. 10.1186/1471-2318-14-14 PMC3924230 24484314

[38] 38. Isles RC, Choy NL, Steer M, Nitz JC. Normal values of balance tests in women aged 20-80. J Am Geriatr Soc. 2004;52(8):1367-72. 10.1111/j.1532-5415.2004.52370.x 15271128

[39] 39. Shumway-Cook A, Brauer S, Woollacott M. Predicting the probability for falls in community-dwelling older adults using the timed up & go test. Phys Ther. 2000;80(9):896-903. 10960937

[40] 40. Li AM, Yin J, Yu CC, Tsang T, So HK, Wong E, et al. The six-minute walk test in healthy children: reliability and validity. Eur Respir J. 2005;25(6):1057-60. 10.1183/09031936.05.00134904 15929962

[41] 41. American Thoracic Society/European Respiratory Society. ATS/ERS Statement on respiratory muscle testing. Am J Respir Crit Care Med. 2002; 166(4):518-624. 10.1164/rccm.166.4.518 12186831

[42] 42. Pohl M, Rockstroh G, Rückriem S, Mrass G, Mehrholz J. Immediate effects of speed-dependent treadmill training on gait parameters in early Parkinson's disease. Arch Phys Med Rehabil. 2003;84(12):1760-6. 10.1016/s0003-9993(03)00433-7 14669180

[43] 43. Shulman LM, Katzel LI, Ivey FM, Sorkin JD, Favors K, Anderson KE, et al. Randomized clinical trial of 3 types of physical exercise for patients with Parkinson disease. JAMA Neurol. 2013;70(2):183-90. 10.1001/jamaneurol.2013.646 PMC4574905 23128427

[44] 44. Carda S, Invernizzi M, Baricich A, Comi C, Croquelois A, Cisari C. Robotic gait training is not superior to conventional treadmill training in parkinson disease: a single-blind randomized controlled trial. Neurorehabil Neural Repair. 2012;26(9):1027-34. 10.1177/1545968312446753 22623206

[45] 45. Toole T, Maitland CG, Warren E, Hubmann MF, Panton L. The effects of loading and unloading treadmill walking on balance, gait, fall risk, and daily function in Parkinsonism. NeuroRehabilitation. 2005;20(4):307-22. 16403997

[46] 46. Ganesan M, Sathyaprabha TN, Gupta A, Pal PK. Effect of partial weight-supported treadmill gait training on balance in patients with Parkinson disease. PM R. 2014;6(1):22-33. 10.1016/j.pmrj.2013.08.604 24021298

[47] 47. Murray MP, Sepic SB, Gardner GM, Downs WJ. Walking patterns of men with parkinsonism. Am J Phys Med Rehab. 1978;57(6):278-94. 742658

[48] 48. Mhatre PV, Vilares I, Stibb SM, Albert MV, Pickering L, Marciniak CM, et al. Wii Fit balance board playing improves balance and gait in Parkinson disease. PM R. 2013;5(9):769-77. 10.1016/j.pmrj.2013.05.019 PMC4114762 23770422

[49] 49. Rubinstein TC, Giladi N, Hausdorff JM. The power of cueing to circumvent dopamine deficits: a review of physical therapy treatment of gait disturbances in Parkinson's disease. Mov Disord. 2002;17(6):1148-60. Review. 10.1002/mds.10259 12465051

