# Supplementary material for: Effects of treadmill training on gait of elders with Parkinson's disease: a literature review
Source: Einstein (Sao Paulo). 2020 Nov 18;18:eRW5233. doi: 10.31744/einstein_journal/2020RW5233 (PMC7687915; doi:10.31744/einstein_journal/2020RW5233)
Supplement: Translation

# Translation: Efeitos do treinamento de marcha em esteira em idosos com doença de Parkinson: uma revisão da literatura

**Authors:** Natália Mariana Silva Luna, Guilherme Carlos Brech, Alexandra Canonica, Rita de Cássia Ernandes, Danilo Sales Bocalini, Julia Maria D’Andréa Greve, Angélica Castilho Alonso

**Affiliations:** 1 São PauloSP Brasil Instituto de Ortopedia e Traumatologia, Hospital das Clínicas, Faculdade de Medicina, Universidade de São Paulo, São Paulo, SP, Brasil.; 2 São PauloSP Brasil Universidade São Judas Tadeu, São Paulo, SP, Brasil.; 3 VitóriaES Brasil Universidade Federal do Espírito Santo, Vitória, ES, Brasil.

**Keywords:** Doença de Parkinson/reabilitação, Marcha, Idoso

## Abstract

A doença de Parkinson é o segundo distúrbio neurodegenerativo mais comum na velhice. O processo de envelhecimento de idosos com doença de Parkinson pode levar a distúrbios de marcha com mais incapacidades funcionais do que para idosos sem a doença. O treinamento em esteira como terapia pode resultar em efeitos notáveis na marcha de pacientes com Parkinson e ser um recurso para a reabilitação neurológica geriátrica. Esta revisão teve como objetivo estudar os efeitos da marcha após o treinamento em esteira na doença de Parkinson em idosos. A pesquisa foi realizada nas bases de dados PubMed®, LILACS, PEDro e EMBASE, com os seguintes descritores: “doença de Parkinson”, “idosos”, “treinamento em esteira” e “avaliação da marcha”. A qualidade dos estudos incluídos foi avaliada pela escala de PEDro. Atenderam aos critérios de inclusão e exclusão 11 estudos. Oito estudos foram randomizados, e apenas um fez *follow-up* . Foi possível observar que treinamento em esteira com ou sem suporte de peso (por pelo menos 20 minutos, duas a três vezes por semana, com aumento progressivo de cargas, por, no mínimo, 6 semanas) em idosos com doença de Parkinson foi efetivo para melhorar a marcha. Além disso, ambos os treinamentos foram considerados seguros (pois alguns estudos relataram o uso de cintos, mesmo no treinamento sem suporte de peso) e podem ser associados a terapias complementares à marcha, como estimulação magnética transcraniana repetitiva, estímulos visuais ou estimulação transcraniana direta anódica. O treinamento em esteira em pacientes idosos com doença de Parkinson é uma intervenção que melhora os resultados da marcha, mas requer mais estudos para melhor comprovação.

## INTRODUÇÃO

A doença de Parkinson (DP) idiopática é crônica, progressiva e degenerativa do sistema nervoso central, estando associada a uma depleção nos neurônios dopaminérgicos na via nigroestriatal.^(^^1^^)^Os sintomas clínicos aparecem quando há redução de 40% a 60% dos neurônios nigrais e da dopamina estriatal.^(^^2^^)^A DP é a segunda doença degenerativa mais comum do sistema nervoso, afetando 1% a 2% da população com mais de 65 anos.^(^^3^^)^A proporção de idosos no mundo está aumentando, o que resulta em maior número de pessoas em risco de DP.^(^^4^^)^

Os sintomas cardinais incluem tremor em repouso, rigidez muscular, congelamento da marcha (FOG - *freezing of gait* ), bradicinesia, e instabilidade de marcha e postural.^(^^3^^,^^4^^)^Os distúrbios da marcha comprometem a independência e a qualidade de vida dos pacientes, diminuindo sua mobilidade e aumentando o risco de quedas e fraturas.^(^^5^^)^A DP induz a distúrbios da marcha, como redução da velocidade, redução do comprimento da passada e do passo, e aumento do tempo de apoio duplo.^(^^4^^–^^8^^)^

A redução da velocidade de marcha é explicada por um deslocamento mais lento do centro de gravidade, um menor componente vertical da força de reação do solo^(^^9^^–^^11^^)^e um maior deslocamento lateral do centro de pressão no início da marcha.^(^^12^^)^A redução no comprimento do passo ocorre principalmente devido à deficiência na fase de descolamento do pé, na qual se observam menor sinal eletromiográfico do músculo gastrocnêmio e consequente dificuldade para projetar o membro para a frente.^(^^10^^)^O aumento da fase de suporte está relacionado à diminuição da velocidade da marcha e à bradicinesia.^(^^5^^)^Isso dificulta o automatismo do passo na marcha e promove alterações no sinal eletromiográfico dos membros inferiores.^(^^13^^,^^14^^)^

Da mesma forma, o envelhecimento também diminui a velocidade da marcha e o comprimento do passo, o que sugere que a DP, em idosos, pode ocasionar maior incapacidade funcional e torná-los mais suscetíveis a quedas.^(^^15^^)^

No tocante à reabilitação física da marcha, o treinamento em esteira (TE) como terapia mostrou efeitos notáveis na marcha de pacientes com DP, com resultados superiores nos parâmetros de marcha em comparação com os pacientes que não são submetidos a esse tratamento.^(^^16^^–^^21^^)^Revisões da literatura mostram que o TE pode promover padrão de caminhada mais estável em pacientes com DP, sugerindo que a intervenção é capaz de restaurar a ritmicidade e reduzir a variabilidade da marcha.^(^^18^^,^^21^^,^^22^^)^No entanto, essas revisões analisam vários desfechos, mas nenhuma enfatiza os idosos.

## OBJETIVO

O objetivo deste estudo foi analisar os efeitos na marcha após treinamento em esteira em pacientes idosos com doença de Parkinson.

## MÉTODOS

### Estratégia de busca

A busca foi realizada nas bases de dados PubMed^®^, LILACS, PEDro e EMBASE, tendo encontrado artigos publicados relacionados aos efeitos do TE na marcha de pacientes idosos com DP, com os seguintes descritores: “doença de Parkinson”, “idosos”, “treinamento em esteira” e “avaliação da marcha”.

### Seleção de estudos

A revisão crítica da literatura foi realizada considerando os seguintes critérios de inclusão: estudos em humanos, no idioma inglês e publicados em periódicos científicos nos últimos 10 anos. Foram excluídos os artigos que descreviam pacientes com idade inferior a 60 anos, ou que não realizaram treinamento de marcha em esteira, e sem medidas de desfecho em relação à marcha. Os artigos que descreviam treinamento de marcha em esteira em pacientes sem DP e treinamento de marcha em esteira combinados com realidade virtual também foram excluídos. Após a leitura dos títulos e/ou resumos, foram selecionados os artigos que atenderam aos critérios de inclusão e identificados como relevantes para o desenvolvimento deste trabalho. Foram encontradas 25 referências após combinações simples no PubMed^®^, LILACS, PEDro e EMBASE. Foram finalmente selecionados 11 artigos com base nos critérios estabelecidos.

### Avaliação da qualidade

A qualidade dos 11 estudos incluídos(23-33) foi avaliada pela lista de verificação da escala PEDro ( Tabela 1 ), que apresenta 11 itens que levam em consideração os seguintes critérios: elegibilidade e origem dos participantes; distribuição randômica; distribuição oculta; comparabilidade da linha de base; cegamento de sujeitos; cegamento de terapeutas; cegamento de avaliadores; resultados com mais de 85% dos participantes; análise de intenção de tratamento; comparações estatísticas entre grupos; e medidas de precisão e medidas de variabilidade.

**Tabela 1:** Escala PEDro de 11 estudos selecionados

Autores	Elegibilidade	Distribuição randômica	Distribuição oculta	Comparabilidade da linha de base	Cegamento de pacientes	Cegamento de terapeutas	Cegamento de avaliadores	Resultados com mais de 85% dos participantes	Análise de intenção de tratamento	Comparações estatísticas entre os grupos	Medidas de precisão e medidas de avaliação
Frazzitta et al.^(^^23^^)^	Sim	Sim	Não	Sim	Não	Não	Sim	Sim	Sim	Sim	Não
Yang et al.^(^^24^^)^	Sim	Não	Sim	Sim	Sim	Sim	Sim	Sim	Sim	Sim	Sim
Galli et al.^(^^25^^)^	Sim	Não	Sim	Sim	Sim	Sim	Sim	Sim	Sim	Sim	Não
Canning et al.^(^^26^^)^	Sim	Não	Sim	Sim	Não	Não	Sim	Sim	Sim	Sim	Sim
Frazzitta et al.^(^^27^^)^	Sim	Sim	Não	Sim	Não	Não	Sim	Sim	Sim	Sim	Não
Picelli et al.^(^^28^^)^	Sim	Não	Sim	Sim	Não	Não	Sim	Sim	Sim	Sim	Não
Fisher et al.^(^^29^^)^	Sim	Não	Sim	Sim	Sim	Não	Não	Sim	Sim	Sim	Não
Schlick et al.^(^^30^^)^	Sim	Não	Sim	Sim	Não	Não	Não	Sim	Sim	Sim	Não
Arcolin et al.^(^^31^^)^	Sim	Não	Não	Sim	Não	Não	Não	Sim	Sim	Sim	Não
Herman et al.^(^^32^^)^	Sim	Não	Não	Não	Não	Não	Não	Sim	Sim	Não	Não
Fernández-Lago et al.^(^^33^^)^	Sim	Não	Não	Sim	Não	Não	Não	Sim	Sim	Sim	Não

## RESULTADOS

Onze estudos atenderam aos critérios de inclusão.^(^^23^^–^^33^^)^Os estudos que obtiveram maior pontuação na escala PEDro foram realizados por Yang et al.,^(^^24^^)^e Galli et al.,^(^^25^^)^( Tabela 1 ).

Dos 11 estudos,^(^^23^^–^^33^^)^oito foram randomizados.^(^^23^^–^^30^^)^Em relação ao tipo de treinamento, cinco estudos analisaram treinamento sem suporte de peso corporal,^(^^23^^,^^24^^,^^26^^,^^27^^,^^30^^)^e três avaliaram treinamento com sistema de suporte^(^^25^^,^^28^^,^^29^^)^( Tabela 2 ).

**Tabela 2:** Resumo sobre população, protocolos, medidas de marcha e resultados destes estudos

Estudos	População	Protocolo	Medidas de marcha	Resultados
Frazzitta et al.^(^^23^^)^	30 pacientes com distúrbio de marcha, mas sem FOG em estágio *on* , e 30 pacientes com FOG em estágio *on*	Os pacientes passaram por tratamento de reabilitação de 4 semanas usando uma esteira com pistas auditivas e visuais	Velocidade da marcha, comprimento da passada, assimetria de marcha, TC6, UPDRS II–III, Escala de Equilíbrio de Berg, TUG, velocidade de marcha confortável, FOG-Q	Os dados apoiam um envolvimento direto da assimetria da marcha no desenvolvimento do FOG na DP. O TE é eficaz para melhoria da marcha e do equilíbrio em pacientes com DP e FOG, e isso pode estar relacionado à redução na assimetria da marcha
Yang et al.^(^^24^^)^	20 pacientes com DP com capacidade de caminhar de forma independente	2 grupos: 1. Participantes que receberam EMTr (Grupo Experimental) 2. Participantes que receberam EMTr simulada (Grupo Controle) Seguido de TE (30 minutos) por 12 sessões durante 4 semanas (3 sessões por semana)	Inibição corticomotora e desempenho da marcha	Os achados sugerem que a combinação de EMTr com TE aumenta o efeito do TE na modulação da inibição corticomotora e na melhoria do desempenho da marcha nos pacientes com DP
Galli et al.^(^^25^^)^	50 pacientes com DP idiopáticaCapacidade de caminhar, sem auxílio ou com pouco auxílio, por distância de 25 pés (7,62m)	2 grupos: 1. Grupo de terapia com assistência robótica (n=25) 2. Grupo de terapia intensiva em esteira (n=25)	Escala Hoehn e Yahr;UPDRSSistema optoeletrônico (ELITE2002, BTS, Milão, Itália)	No grupo robótico, foram encontradas diferenças nas variáveis cinemáticas (obliquidade pélvica e abdução e adução do quadril). O grupo intensivo não mostrou alterações estatisticamente significativas. O treinamento locomotor de reabilitação robótica para efetor final melhorou as características cinemáticas da marcha e pareceu ser eficaz na reabilitação de pacientes com DP leve
Canning et al.^(^^26^^)^	20 pacientes com DP idiopática com <2 horas por semana de atividade física de lazer nos 3 meses anteriores e que tiveram resposta estável à levodopa	2 grupos: 1. O Grupo Experimental foi submetido a um programa semissupervisionado de 30 a 40 minutos de caminhada na esteira, realizado no lar, 4 vezes por semana, durante 6 semanas 2. O Grupo Controle recebeu os cuidados habituais (ou seja, foi aconselhado a manter os níveis atuais de atividade física)	O desfecho primário de eficácia foi a capacidade de caminhar (distância percorrida no TC6)	O TE semissupervisionado no lar é uma forma viável e segura de exercício para pessoas cognitivamente intactas, com DP leve
Frazzita et al.(27)	40 pacientes parkinsonianos com congelamento foram aleatoriamente designados a dois grupos	2 grupos: 1. Foi submetido a um programa de reabilitação baseado em treinamento em esteira associado a pistas auditivas e visuais 2. Seguiu um protocolo de reabilitação usando dicas e não associado a esteira	A avaliação funcional foi baseada na *Unified Parkinson's Disease Rating Scale Motor Section* (UPDRS III); *Freezing of Gait Questionnaire* (FOGQ), teste de caminhada de 6 minutos (TC6), velocidade da marcha e ciclo de passada	Os resultados sugerem que o treinamento em esteira associado a pistas auditivas e visuais pode dar melhores resultados do que os tratamentos convencionais. O treinamento em esteira provavelmente atua como uma dica externa complementar
Picelli et al.^(^^28^^)^	60 pacientes com DP leve a moderada	- 3 grupos: 1. Grupo de treinamento de marcha com assistência robótica (n=20) 2. Grupo de TE (n=20) realizou treinamento em intensidade igual sem apoio de peso corpóreo 3. Grupo de fisioterapia (n=20) foi submetido à terapia convencional da marcha45 minutos por sessão 3 dias por semana, por 4 semanas consecutivas	Teste de caminhada de 10 minutos TC6	Nenhuma diferença estatisticamente significativa foi encontrada entre o grupo de treinamento de marcha com assistência robótica e o grupo de TE na avaliação após o treinamento. Uma melhora estatisticamente significativa foi encontrada após o tratamento, a favor do grupo de treinamento de marcha com assistência robótica e do grupo de TE, em comparação com o grupo de fisioterapia. Os achados foram confirmados na avaliação de acompanhamento de 3 meses
Fisher et al.^(^^29^^)^	30 pacientes com DP, dentro de 3 anos após o diagnóstico, com estágios 1 ou 2 na escala de Hoehn e Yahr	- 3 grupos: 1. Alta intensidade (TE com apoio de peso corporal) 2. Exercício de baixa intensidade (exercícios para amplitude de movimento, equilíbrio e marcha) 3. Intensidade zero (grupo de orientação)	UPDRS Análise biomecânica da caminhada autosselecionada e da caminhada rápida Tarefas sentado para de pé Excitabilidade corticomotora	Foi observada pequena melhora no UPDRS total e motor em todos os grupos. Os indivíduos do grupo de alta intensidade mostraram aumento pós-exercício na velocidade da marcha, no comprimento do passo e da passada, e na excursão das articulações do quadril e do tornozelo, durante a marcha autosselecionada e a marcha rápida, além de melhor distribuição do peso nas tarefas sentado para de pé. Não foi observada melhora consistente nas medidas da marcha e nas tarefas sentado para de pé nos grupos de baixa intensidade e de intensidade zero. O grupo de alta intensidade apresentou aumento no período de silêncio cortical
Schlick et al.^(^^30^^)^	23 pacientes ambulatoriais com DP	Os pacientes foram submetidos a 12 sessões de treinamento dentro de 5 semanas com pistas visuais combinadas ao TE (n=12) ou apenas TE (n=11)	Velocidade da marcha, comprimento da passada e cadência; Os testes funcionais incluíram o TUG, UPDRS e o questionário de congelamento da marcha	Este estudo piloto sugere que as pistas visuais combinadas ao TE têm efeitos mais benéficos na marcha do que o TE, em pacientes com estágio moderado da DP. Faz-se necessário um estudo em larga escala com acompanhamento mais longo
Arcolin et al.^(^^31^^)^	29 pacientes com diagnóstico de DP idiopáticaHabilidade para caminhar, sem ajuda	2 grupos (randomizados): 1. Esteira (n=13) 2. Cicloergômetro (n=16) Treinamento por 3 semanas, 1 hora por dia	TC6 Variáveis espaciais e temporais da marcha avaliadas por baropodometria TUG MINIBESTest UPDRS	Este estudo piloto mostra que o treinamento em cicloergômetro melhora os parâmetros de caminhada e reduz os sinais clínicos de DP, tanto quanto o TE. A velocidade da marcha é acompanhada pelo aumento no comprimento do passo, tornando o padrão da marcha próximo ao de indivíduos saudáveis. O cicloergômetro é alternativa válida à esteira para melhorar a marcha em curto prazo em pacientes com DP
Herman et al.^(^^32^^)^	9 pacientes com DP que eram capazes de deambular de forma independente e não estavam demenciados	Os pacientes caminharam em esteira por 30 minutos durante cada sessão de treinamento, em 4 sessões de treinamento por semana, durante 6 semanas	PDQ-39, com 39 perguntasUPDRSVelocidade da marcha, variabilidade no tempo da passada, variabilidade no tempo de balançoSPPB	Esses resultados mostram o potencial para aumento da ritmicidade da marcha em pacientes com DP e sugerem que um programa de TE progressivo e intensivo pode ser usado para minimizar défices de marcha, reduzir o risco de queda e aumentar a qualidade de vida desses pacientes
Fernández-Lago et al.^(^^33^^)^	18 pacientes com DP idiopática Habilidade de caminhar, sem ajuda	Foram avaliados 18 participantes com DP sob estas três condições (grupos):1. Somente caminhada em esteira (esteira) 2. Caminhada em esteira combinada a ETCC anódica (ETCCa + esteira) aplicada ao córtex motor;3. Caminhada em esteira combinada a estimulação simulada (ETCCs + esteira).	Desempenho de caminhada no solo, reflexo H do músculo sóleo, inibição recíproca da fibra sensorial Ia do músculo tibial anterior para o músculo sóleo, facilitação intracortical e inibição intracortical de curta duração do músculo tibial anterior	Todas as configurações de esteira melhoraram o desempenho da caminhada e modularam os parâmetros espinais e corticospinais comparando-se a outros tipos de tratamento. No entanto, a opção ETCCa + esteira resultou em modulação diferente da inibição recíproca da fibra sensorial Ia, em comparação com as outras configurações de esteira

FOG: congelamento da marcha; TC6: teste de caminhada de 6 minutos; UPDPRS: Escala Unificada de Avaliação da Doença de Parkinson; TUG: Timed Up and Go; FOG-Q: Questionário de Congelamento da Marcha; TE: treinamento em esteira; DP: doença de Parkinson; EMTr: estimulação magnética transcraniana repetitiva; MINIBESTest: *Balance Evaluation Systems Test* ; PDQ-39: Parkinson Disease Questionnaire-39; SPPB: Short Physical Performance Battery; ETCC: estimulação transcraniana por corrente contínua.

## DISCUSSÃO

Alguns estudos sobre TE em pacientes com DP, assim como revisões da literatura, abordam o assunto. No entanto, as revisões analisam grupos de todas as idades e apresentam metodologias com desfechos primários diferentes: qualidade de vida, equilíbrio e marcha. Nossa revisão teve como objetivo focar apenas estudos com grupos de pacientes com DP com mais de 60 anos, bem como desfechos relacionados a especificidades da marcha. De acordo com a lista de verificação da escala PEDro, os artigos incluídos foram considerados adequados para publicação científica.

A escala Hoehn e Yarh variou de 1 a 3, e a maioria dos pacientes^(^^23^^,^^25^^–^^27^^,^^29^^,^^32^^,^^33^^)^estudados estava entre 1 e 2 – classificação essa que indica discreto *deficit* de equilíbrio. Em quatro estudos,^(^^24^^,^^28^^,^^30^^,^^31^^)^o estágio estava entre 2 e 3, uma classificação na qual a doença é obrigatoriamente bilateral e há certa instabilidade postural, mas o paciente é independente em termos físicos.

A escala Hoehn e Yarh caracteriza a condição funcional do paciente por meio de observação.^(^^34^^)^Outros estudos com pacientes com menos de 60 anos ou que mesclam diferentes grupos etários mostraram essa pontuação da escala Hoehn e Yarh. Assim, a classificação Hoehn e Yarh moderada pode sugerir que o envelhecimento não interferiu na doença.

A duração da doença foi outra variável demográfica que diferiu nos estudos. Em oito estudos,^(^^15^^–^^17^^,^^19^^,^^21^^–^^24^^)^a duração ficava entre 5 e 10 anos e, em dois, a duração foi de mais de 10 anos.^(^^27^^,^^29^^)^Somente um estudo não mencionou esse dado.^(^^23^^)^Os estudos mostraram que, com a evolução da doença (5 a 7 anos), há piora da condição motora e surgimento de discinesias.^(^^15^^)^Dentre os pacientes que utilizaram levodopa por 6 anos ou mais, 70% tiveram complicações motoras e precisaram suspender temporariamente seu uso ( *off* ).^(^^35^^)^

As avaliações da marcha utilizadas nos estudos de revisão foram computadorizadas ou funcionais. Ambas são importantes para a explicação dos resultados, mas algumas são mais específicas que outras e, portanto, podem ajudar mais na obtenção de respostas para as questões encontradas na prática clínica.^(^^11^^)^

A avaliação motora da Escala Unificada de Avaliaçãoda Doença de Parkinson (UPDRS) foi utilizada na maioria dos estudos.^(^^23^^,^^29^^–^^32^^)^É uma escala muito confiável (r=0,96) e válida, constituindo método adequado para a avaliação da DP.^(^^36^^)^A seção de avaliação motora consiste em 14 itens (18 a 31), mas o item que avalia a marcha é de natureza observacional, não permitindo análise precisa.

O teste *Timed Up and Go* (TUG) também foi utilizado em cinco estudos.^(^^23^^,^^27^^,^^30^^–^^32^^)^O TUG mede o tempo da tarefa motora de se levantar da cadeira, andar 3m, retornar e sentar-se.^(^^37^^)^Isles et al.,^(^^38^^)^definem o teste como sensível e específico para discriminar entre caidores e não caidores. É possível observar que o teste é utilizado para avaliar o equilíbrio e a mobilidade^(^^39^^)^sem, porém, analisar características específicas da marcha.

Outra ferramenta de avaliação utilizada foi o teste de caminhada de 6 minutos (TC6).^(^^14^^,^^16^^–^^19^^)^Esse teste é utilizado para avaliar a resposta física e fornece análise abrangente dos sistemas respiratório, cardíaco e metabólico.^(^^40^^)^Ele reflete limitações nas atividades cotidianas, como a marcha, mas, de acordo com a *American Thoracic Society* (ATS),^(^^41^^)^a indicação precisa do TC6 é a presença de doença pulmonar ou doença cardíaca leve a moderada, de modo que o teste é usado para medir a resposta ao tratamento e prever a morbimortalidade.

Os parâmetros espaciais (velocidade, comprimento do passo e cadência) são considerados significativos para a avaliação clínica da marcha. Eles podem ser calculados qualitativamente, sem câmeras nem *softwares* , ou de modo computadorizado, por meio de análises cinemáticas e cinéticas. Cinco estudos investigaram esses parâmetros por abordagem qualitativa^(^^27^^,^^29^^–^^32^^)^e apenas dois o fizeram por meios computadorizados.^(^^25^^,^^29^^)^O uso da análise tridimensional da marcha é escasso nos estudos sobre DP, mas é considerado o método padrão-ouro para melhor compreensão biomecânica, fornecendo dados reproduzíveis e confiáveis.^(^^13^^)^

O TE foi estudado com e sem suporte do peso corporal. Oito estudos analisaram o treinamento sem suporte do peso corporal,^(^^14^^–^^18^^,^^22^^–^^24^^)^e três avaliaram com sistema de suporte.^(^^19^^–^^21^^)^Todos apresentaram melhora nas variáveis da marcha após o treinamento, mas houve diferenças entre as metodologias.

Somente Herman et al.,^(^^32^^)^realizaram estudo de acompanhamento. Realizaram seis semanas de TE (quatro vezes por semana, 30 minutos cada sessão), seguido de 1 mês de TE com aumento progressivo da intensidade em nove pacientes com DP. Houve melhora do UPDRS no tocante à velocidade e ao comprimento do passo, além de variabilidade da marcha após período de treinamento e acompanhamento de 6 semanas. Esses resultados sugeriram que um programa progressivo e intensivo de TE pode ser usado para minimizar deficiências na marcha, reduzir o risco de queda e aumentar a qualidade de vida desses pacientes.

Frazzitta et al.,^(^^23^^)^recrutaram pacientes com Parkinson com e sem FOG, que foram submetidos ao mesmo protocolo de TE sem suporte associado a pistas visuais e auditivas. Os indivíduos receberam TE por 30 minutos todos os dias, 5 dias por semana, durante 4 semanas (20 sessões no total). O TE foi eficaz para melhorar a marcha e o equilíbrio em pacientes com DP, e essa assimetria na marcha pode estar relacionada a uma maior probabilidade de FOG.

Os outros compararam o TE tradicional com outro tipo de TE. Frazzitta et al.,^(^^27^^)^compararam o TE sem suporte associado a pistas visuais e auditivas com o treinamento de marcha sem esteira, também associado a pistas visuais e auditivas. Os resultados sugerem que o TE associado a pistas auditivas e visuais pode produzir melhores resultados do que os tratamentos mais convencionais. Os indivíduos receberam treinamento por 20 minutos todos os dias, durante 4 semanas (28 sessões no total). O TE provavelmente atua como pista externa suplementar.

Schlick et al.,^(^^30^^)^analisaram 20 pacientes com DP, que foram divididos em duas intervenções: grupo TE, com pistas visuais, e grupo TE, sem pistas. Esse estudo piloto sugere que as pistas visuais combinadas com o TE têm efeitos mais benéficos na marcha do que o TE tradicional. As pistas visuais atuam como estímulo para os receptores visuais, gerando estímulos aferentes sensoriais repetitivos para o sistema nervoso central. Em pacientes com DP, esses sinais também podem representar pistas sensoriais externas, sendo um gatilho para a ativação de circuitos intactos não afetados pela doença, como a via para o córtex pré-motor lateral.^(^^2^^,^^6^^)^

Yang et al.,^(^^24^^)^compararam o TE com e sem aplicação prévia de estimulação magnética transcraniana repetitiva (EMTr). O TE foi realizado por 12 sessões (3 sessões por semana) ao longo de um período de 4 semanas. Todos os participantes caminharam em uma esteira motorizada (Biodex, Shirley, New York, USA) após a EMTr. Os resultados sugeriram que a combinação de EMTr e TE aumenta o efeito do TE na modulação da inibição corticomotora e na melhora do desempenho da marcha em pacientes com DP.

Canning et al.,^(^^26^^)^observaram um Grupo Experimental que foi submetido a um programa semissupervisionado de 30 a 40 minutos de caminhada na esteira, realizado no lar, quatro vezes por semana, durante 6 semanas. O Grupo Controle recebeu os cuidados habituais (ou seja, conselhos para manter o nível de atividade física atual). O TE semissupervisionado em casa foi uma forma viável e segura de exercício para pessoas cognitivamente intactas com DP leve. O tratamento no lar é uma estratégia importante na reabilitação geriátrica, sendo possível associar o TE como reforço aos exercícios de marcha.

Arcolin et al.,^(^^31^^)^randomizaram 29 pacientes com DP internados para TE (n=13) ou com cicloergômetro (n=16) por 3 semanas, 1 hora por dia. Esse estudo piloto mostra que o treinamento com cicloergômetro melhora os parâmetros de marcha e reduz os sinais clínicos de DP, tanto quanto o TE. A velocidade da marcha é acompanhada pelo aumento no comprimento do passo, tornando o padrão da marcha próximo do de indivíduos saudáveis. O cicloergômetro é uma alternativa válida à esteira, para melhorar a marcha a curto prazo em pacientes com DP.

Os estudos que não foram incluídos na revisão porque a amostra não era de idosos também mostraram que o TE sem suporte de peso corporal em pacientes com DP, em comparação com outras terapias motoras, aumentou a velocidade e o comprimento da passada.^(^^42^^,^^43^^)^

Três estudos incluídos utilizaram TE com suporte de peso,^(^^18^^–^^20^^)^sendo que dois^(^^19^^,^^21^^)^mostraram superioridade do treinamento com suporte em comparação com outras terapias, e um estudo não observou diferença.^(^^28^^)^Fisher et al.,^(^^29^^)^recrutaram 30 pacientes com DP (estágio Hoehn e Yarh I a II) que foram randomizados em três grupos: terapia de alta intensidade (treinamento com suporte), fisioterapia de baixa intensidade (amplitude de movimento passiva e ativa, equilíbrio, marcha, resistência muscular e exercícios de funções diárias) e terapia não intensiva, caracterizada por educação terapêutica (seis aulas sobre qualidade de vida durante período de 8 semanas). As intervenções duraram 8 semanas, totalizando 24 sessões (três vezes por semana com sessões de 1 hora). O TE iniciou com 10% do peso corporal suspenso. No entanto, se o paciente não conseguisse realizar a marcha, esse percentual foi aumentado, de modo que o objetivo de cada sessão era o de atingir e manter um nível superior a 3 METs, respeitando o repouso, quando necessário. Os resultados mostraram melhora do UPDRS em todos os grupos. O grupo de treinamento com suporte mostrou aumento na velocidade da marcha, no comprimento do passo e da passada e na excursão do quadril e do tornozelo no plano sagital, além de melhor distribuição do peso durante o exercício e aumento do período de silêncio cortical.

Gali et al.,^(^^25^^)^e Picelli et al.,^(^^28^^)^também utilizaram treinamento com suporte do peso corporal, porém com assistência robótica. Picelli et al.,^(^^28^^)^compararam três grupos: o grupo de treinamento de marcha com assistência robótica, o grupo de TE sem suporte do peso corporal e o grupo de fisioterapia. Cada sessão de treinamento compreendia três partes, com descanso de 5 minutos após cada uma. A princípio, os pacientes foram treinados a uma velocidade de 1km/hora, por 10 minutos; depois, a uma velocidade de 1,5km/hora, por 10 minutos; e, por fim, a uma velocidade de 2,0km/hora, por 10 minutos. Não houve diferenças nos parâmetros espaciais e temporais dos testes de 6 e 10 minutos de caminhada. Gali et al.,^(^^25^^)^avaliaram 50 pacientes com DP, que foram divididos em dois grupos: 25 foram designados para a terapia com assistência robótica e 25 para o grupo de terapia intensiva em esteira. O treinamento locomotor da reabilitação com assistência robótica do efetor final melhorou a cinemática da marcha, e parece ser eficaz para a reabilitação de pacientes com DP leve.

Alguns estudos com pacientes não idosos também observaram resultados semelhantes. Não houve diferença nas variáveis temporais e espaciais dos testes de caminhada de 6 e 10 minutos e do teste TUG e do UPDRS,^(^^44^^)^quando comparados o TE e o TE Lokomat. Outros estudos observaram o efeito do TE com suporte de peso em relação a sem suporte de peso,^(^^45^^)^ao treinamento de marcha no solo^(^^46^^)^e a outras terapias motoras.^(^^16^^,^^17^^)^

Toole et al.,^(^^45^^)^relataram os efeitos do TE por 6 semanas, em 23 pacientes com DP, randomizados em três grupos. Dois grupos treinaram a marcha com 25% e 5% de suporte do peso corporal, e o terceiro grupo sem suporte. Os três grupos melhoraram nas escalas UPDRS e Berg, sem diferença entre eles. O estudo de Ganesan et al.,^(^^46^^)^utilizou posturografia computadorizada para avaliar o efeito de um TE de 4 semanas com suporte de 20% do peso corporal, quatro vezes por semana (30 minutos por dia). Os autores relataram melhoria nos limites de estabilidade e na oscilação lateral medial do centro de massa com o treinamento com suporte em relação ao treinamento de marcha convencional fora da esteira. Miyai et al.,^(^^16^^)^compararam o treinamento de marcha com 20% de suporte do peso corporal em relação à fisioterapia convencional por 4 semanas em pacientes com DP e relataram que o TE com suporte de peso melhorou o desempenho motor da marcha (teste de caminhada de 10 minutos) e as atividades da vida diária, que persistiram por 6 meses.^(^^17^^)^

Fernández-Lago et al.,^(^^33^^)^descreveram que uma única sessão combinando caminhada na esteira e estimulação transcraniana por corrente contínua (ETCC) aplicada sobre o córtex motor resultou em modulação específica da inibição recíproca da fibra sensorial Ia do músculo tibial anterior ao músculo sóleo. No entanto, esse efeito agudo não resultou em melhora dos parâmetros da marcha associados à caminhada na esteira na DP.

O treinamento sem suporte do peso corporal parece ser mais difícil, pois exige maior demanda muscular. As principais justificativas para essa evidência são velocidade constante; estimulação sensorial contínua; pistas sensoriais externas; ativação de circuitos neurais da marcha gerando padrão central; *feedback* visual; velocidade constante e aprendizado motor.^(^^16^^,^^17^^,^^45^^,^^46^^)^

O treinamento com suporte do peso corporal, mesmo que diminua a estimulação periférica (força muscular e esforço aeróbico), pode aumentar a estimulação proprioceptiva.^(^^47^^)^O suporte parcial do peso também pode prolongar o período de treinamento útil, pois reduz a fadiga.^(^^48^^)^Além disso, facilita o movimento dos membros inferiores^(^^49^^)^e, portanto, pode ser considerado uma intervenção promissora para o treinamento do controle sensório-motor por meio da melhoria dos reflexos posturais.^(^^16^^,^^17^^)^Também pode facilitar o uso de estratégias que utilizam vias não dopaminérgicas e não são afetadas pela doença. Outros autores sugerem que o treinamento com suporte de peso estimula o centro medular de controle da marcha.^(^^17^^,^^47^^)^

É possível observar em revisão que o TE com ou sem suporte de peso (pelo menos 20 minutos, duas a três vezes por semana, com aumento progressivo das cargas, por, no mínimo, 6 semanas) em pacientes idosos com DP foi eficaz para melhorar a marcha. Além disso, ambos são considerados seguros (uma vez que, mesmo no treinamento sem suporte, os estudos descreveram o uso de cintos) e podem ser associados a terapias complementares à marcha, como EMTr, pistas visuais ou estimulação transcraniana por corrente contínua anódica (ETCCa).

Porém, é importante enfatizar que as avaliações utilizadas para a marcha diferiam entre si. Cinco estudos^(^^17^^,^^19^^,^^21^^,^^22^^,^^24^^)^foram capazes de avaliar as características clínicas da marcha, o que pode auxiliar na evolução do tratamento. Apenas dois usaram a medida padrão-ouro: a avaliação computadorizada.^(^^25^^,^^29^^)^O TE com ou sem suporte de peso pode ser uma intervenção para a reabilitação neurológica geriátrica, mas há necessidade de mais estudos sobre esse tema, que caracterizem melhor a amostra de idosos com DP, além de utilizar ferramentas de avaliação mais específicas da marcha.

Este estudo teve algumas limitações. Não analisamos os subgrupos de pacientes idosos nos estudos que recrutavam adultos na amostra, os quais foram excluídos. A maioria dos estudos tinha amostra relativamente pequena, com total de 300 pacientes em todos os estudos.

## CONCLUSÃO

O treinamento em esteira com ou sem suporte do peso corporal (no mínimo 20 minutos, duas a três vezes por semana, com aumento progressivo da carga, por pelo menos 6 semanas) é uma intervenção que melhora os desfechos da marcha em pacientes idosos com doença de Parkinson, porém se faz necessária a realização de mais estudos.
